# High frequency of Y chromosome microdeletions in male infertility patients with 45,X/46,XY mosaicism

**DOI:** 10.1590/1414-431X20198980

**Published:** 2020-02-14

**Authors:** Leilei Li, Han Zhang, Yi Yang, Hongguo Zhang, Ruixue Wang, Yuting Jiang, Ruizhi Liu

**Affiliations:** Center for Reproductive Medicine and Center for Prenatal Diagnosis, The First Hospital of Jilin University, Changchun, Jilin, China

**Keywords:** 45,X/46,XY mosaicism, Y chromosome microdeletions, Male infertility, Azoospermia, Oligozoospermia

## Abstract

The mosaic 45,X/46,XY karyotype is a common sex chromosomal abnormality in infertile men. Males with this mosaic karyotype can benefit from assisted reproductive therapies, but the transmitted abnormalities contain 45,X aneuploidy as well as Y chromosome microdeletions. The aim of this study was to investigate the clinical and genetic characteristics of infertile men diagnosed with 45,X/46,XY mosaicism in China. Of the 734 infertile men found to carry chromosomal abnormalities, 14 patients were carriers of 45,X/46,XY mosaicism or its variants, giving a prevalence of 0.27% (14/5269) and accounting for 1.91% (14/734) of patients with a chromosomal abnormality. There were ten cases (71.43%, 10/14) of 45,X mosaicism exhibiting AZF microdeletions. Case 1 and Case 4 had AZFc deletions, and the other eight cases had AZFb+c deletions. A high frequency of Y chromosome microdeletions were detected in male patients with 45,X/46,XY mosaicism. Preimplantation genetic diagnosis should be offered to men having intracytoplasmic sperm injection for hypospermatogenesis caused by 45,X/46,XY mosaicism, to avoid the risk of transfering AZF microdeletions in addition to X monosomy in male offspring.

## Introduction

The three main genetic factors known to cause male infertility are cystic fibrosis gene mutations that cause obstructive azoospermia, and chromosomal abnormalities and Y chromosome microdeletions that cause spermatogenic impairment (1). Infertile patients with severe spermatogenic impairment have an increased proportion of genetic abnormalities. The mosaic 45,X/46,XY karyotype is a common sex chromosomal abnormality associated with male infertility.

The detection rate of 45,X/46,XY mosaicism, which is a rare chromosomal abnormality and usually not diagnosed in time, is 1.5/10,000 newborns (2). The clinical phenotype of 45,X/46,XY individuals is very broad and includes Turner females, varying degrees of genital malformations, and men with normal phenotypes (3). The gonads of such patients have been reported as streak gonads, ovarian-like, and/or exhibiting other histopathological abnormalities in previous case reports, small studies, and large multicenter histological studies (4,5).

The 45,X/46,XY mosaic patients are usually infertile and can be identified by karyotype detection. Recently, a close relationship between Y chromosome microdeletions and 45,X/46,XY mosaicism has been identified in Turner’s syndrome patients, patients with mixed gonadal dysgenesis (MGD), and males with infertility. Research also suggested that Y chromosome instability due to Y chromosome microdeletions is more notable in gonadal cells (6–8).

Males with the mosaic 45,X/46,XY karyotype can benefit from assisted reproductive therapies, but prenatal diagnosis plays an important role in preventing the potential transmission of genetic abnormalities. Such transmitted abnormalities contain the 45,X aneuploidy as well as Y chromosome microdeletions. The aim of our study was to investigate the clinical and genetic characteristics of infertile individuals diagnosed with 45,X/46,XY mosaicism in China.

## Material and Methods

### Patients

Five thousand two hundred and sixty-nine cases of primary infertility in men aged 22–46 years were collected from July 7, 2008 to April 21, 2018 at the Center for Reproductive Medicine of the First Hospital of Jilin University. The duration of infertility in these patients ranged from 1 to 11 years. The clinical examination results and family history, gestation history, adverse environmental exposure history, and lifestyle habits of these patients were collected for etiological analysis. The exclusion criteria were infertility caused by female factors, anatomical factors, infectious factors, immune factors, and other known infertility factors. The inclusion criterion was men with infertility considered to be caused by genetic factors other than the exclusion factors. The study protocol was approved by the Ethics Committee of the First Hospital of Jilin University, and written informed consent was obtained from all participants.

### Cytogenetic analysis

All patients were screened by cytogenetic analysis. Peripheral blood (0.5 mL) was collected in sterile tubes containing 30 U/mL heparin. Lymphocytes were cultured in appropriate culture media (Yishengjun; Guangzhou Baidi Biotech, China) for 72 h, and then treated with 20 µg/mL colcemid for 1 h. G-banding of metaphase chromosomes and karyotype analysis were performed using our previously published methods (9).

### Detection of AZF microdeletions

AZF microdeletions were detected by multiplex polymerase chain reaction (PCR). Ten specific sequence-tagged sites, including SY84 and SY86 for AZFa, SY127, SY134, and SY143 for AZFb, and SY152, SY157, SY254, and SY255 for AZFc, were collected according to the European Academy of Andrology and European Molecular Genetics Quality Network. Sex-determining region Y (SRY) and human zinc-finger protein-encoding genes (ZFX/ZFY) located on the X and Y chromosomes were selected as internal control primers (10). Two milliliters of peripheral blood was extracted from each patient with EDTA anticoagulant and then genomic DNA was separated using a blood DNA extraction mini kit (Beijing Tiangen Biotech Co., Ltd., China).

Multiplex PCR amplification was performed using an Amplitron II PCR apparatus (Barnstead/Thermolyne, USA) and 10 µL reaction solutions containing 200 ng of genomic DNA, 1.5 mmol/L Mg^2+^, 800 µmol/L deoxyribonucleotide triphosphates, 10 pmol/L of each primer, and 2 U Taq polymerase. Thermocycling (Veriti Thermal Cycler 96-well, Alpha-SE, Applied Biosystems, USA) consisted of denaturation for 6 min at 94°C, followed by 35 cycles of 94°C for 40 s, 55°C for 45 s, and 72°C for 60 s, with a final extension at 72°C for 6 min. After completion of cycling, the PCR product was removed and mixed with 2 µL of 6× buffer solution, then loaded onto a 1.5% agarose gel containing 0.5 µg/mL ethidium bromide. Electrolysis was performed at 80 V for 35 min, and results were observed using an ultraviolet transmissometer. Samples with detected AZF microdeletions were repeated to verify the results. Samples from a fertile man and woman were used as positive and negative controls, respectively, and water was used as a blank control.

## Results

A total of 734 carriers with chromosomal abnormalities were detected in 5,269 male patients with primary infertility. Of 734 carriers of chromosomal abnormalities, 14 patients were carriers of a 45,X/46,XY mosaicism or its variants, as shown in Supplementary Table S1, giving a prevalence of 0.27% (14/5269) in patients with male infertility and accounting for 1.91% (14/734) of patients with chromosomal abnormalities. Of these 14 patients with mosaicism, 42.86% (6/14) were mosaic for a structurally abnormal Y chromosome in the 46,XY cell line. Eight of the 14 patients (57.14%) had pure 45,X and 46,XY mosaicism. One (1/14, 7.14%) of these patients exhibited oligozoospermia, while the remaining 13 patients exhibited azoospermia. The results of karyotype and AZF microdeletion analysis for the 14 patients are summarized in Supplementary Table S1.

We screened the 14 cases for Y chromosome microdeletions by PCR using 10 Y chromosome-specific markers. Ten cases (71.43%, 10/14) of the 45,X mosaicism exhibited AZF microdeletions. Case 1 and Case 4 represented AZFc deletions, and the other eight cases had AZFb+c deletions. Cases 5 and 6 exhibited a structurally abnormal Y chromosome with no microdeletions detected in the AZF region. The remaining two cases with no AZF deletion had a mosaic of 45,X of 19 and 60%.

## Discussion

The prevalence of 45,X/46,XY mosaicism or its variants in this male infertility study was 0.27% (14/5269), which is much higher than its incidence in newborns. This mosaicism was an important genetic factor for spermatogenesis failure. An important finding of this study was the identification of the very high frequency (71.43%) of Y chromosome microdeletions in patients with sex chromosome mosaicism (45,X/46,XY). Y chromosome microdeletions are one of the main causes of male infertility. It occurs in about 1 in 4000 men in the general population, but its frequency is significantly higher in infertile men. According to global estimates, about 10% of cases of idiopathic infertility occur due to deletions in the AZF region. The detection rate of AZF microdeletion in 45,X/46,XY patients can relate to the clinical phenotype, with different detection rates for the differing phenotypes, but it is basically maintained in a stable range. One previous study found an AZF deletion frequency of approximately 40% in 45,X/46,XY mosaicism patients who presented with partial and mixed gonadal dysgenesis (6), whereas Patsalis et al. reported a deletion incidence rate of 50% in four male patients with azoospermia or mixed gonadal dysgenesis (7). In three other reports, 10 patients with male infertility showed a 100% frequency of Y chromosome deletions (8,11,12). The prevalence of AZF deletions in patients with 45, X/46,XY mosaicism was lower in our study compared with other reports of the same clinical phenotypes, and this may reflect the larger sample size in our study (Table 1, refs. 6–8,11–16).

**Table 1:** Frequency of AZF microdeletions in men with 45,X/46,XY mosaicism or its variants reported in the literature.

Reference	Number of patients	Clinical features	Frequency of AZF microdeletions on peripheral blood lymphocytes
Siffroi et al., 2000 (8)	6	Azoospermia	100% (6/6)
Patsalis et al., 2002 (14)	12	Sexual ambiguities or characteristic of Turner syndrome	33% (4/12)
Papadimas et al., 2001 (13)	1	Ambiguous genitalia	100% (1/1)
Alvarez-Nava et al., 2008 (15)	11	Gonadal dysgenesis	27% (3/11)
Cui et al., 2007 (11)	1	Azoospermia	100% (1/1)
dos Santos et al., 2013 (6)	15	Partial and mixed gonadal dysgenesis	40% (6/15)
Alvarez-Nava et al., 2006 (16)	2	Pure gonadal dysgenesis and Turner syndrome phenotype or mixed gonadal dysgenesis	0 (0/2)
Bettio et al., 2006 (12)	3	Infertility	100% (3/3)
Patsalis et al., 2005 (7)	4	Azoospermia or mixed gonadal dysgenesis	50% (2/4)
Current study	14	Azoospermia or oligozoospermia	71.43% (10/14)

We noted that the 45,X/46,XY mosaic patients with AZF microdeletion may have a vertical inheritance of the Y chromosome carrying AZF deletions from their father. This possibility was supported by a report that described a 17-year-old boy with ambiguous genitalia who had a 45,X/46,XY mosaic karyotype and AZF microdeletions (13 [Bibr B14] [Bibr B15] [Bibr B16]). These findings suggested that fathers who carry AZF microdeletions would transfer a defective Y chromosome to male offspring through natural or assisted reproductive technologies, which may also greatly increase the risk of offspring containing sex chromosome aneuploidy. This risk is currently difficult to assess because it includes not only 45,X/46,XY individuals in this study, but also those with X monosomy.

The structural aberrations of the Y chromosome on the karyotype is often associated with the Y chromosome aneuploidy. A previous report showed that more than half of the patients with Y chromosome structural abnormalities may present with 45,X mosaic cell lines (8). In the present study, 42.86% (6/14) of the mosaic patients were mosaic for a structurally abnormal Y chromosome in 46,XY cell lines; three with Yqh-, one with a Yq deletion, one with a Yp+, and the last with a dicentric Y. Four of these patients also presented with AZF microdeletions.

In conclusion, we found a high frequency of Y chromosome microdeletions in male patients with 45,X/46,XY mosaicism. The percentage of the detected 45,X cell population was not related to the severity of the symptoms. To quantify the potential risk, we recommend that preimplantation diagnosis should be offered when men have intracytoplasmic sperm injection for hypospermatogenesis caused by 45,X/46,XY mosaicism, to avoid the transfer of AZF microdeletions in addition to 45,X in male offspring.

## Supplementary Material

Click here to view [pdf].
